# Supplementary material for: A Comprehensive Assessment of Ultraviolet-Radiation-Induced Mutations in Flammulina filiformis Using Whole-Genome Resequencing
Source: J Fungi (Basel). 2024 Mar 20;10(3):228. doi: 10.3390/jof10030228 (PMC10971301; doi:10.3390/jof10030228)
Supplement: Supplementary file 1 [file jof-10-00228-s001.zip › Supplementary Material S8/KEGG annotation/out/64381550635650.os/KO/out_map/map04330.html]

KEGG PATHWAY: Notch signaling pathway - Reference pathway


|  |  |
| --- | --- |
| **Notch signaling pathway - Reference pathway** |  |

[
Pathway menu
| Organism menu
| Pathway entry
| Show description
| User data mapping
]

The Notch signaling pathway is an evolutionarily conserved, intercellular signaling mechanism essential for proper embryonic development in all metazoan organisms in the Animal kingdom. The Notch proteins (Notch1-Notch4 in vertebrates) are single-pass receptors that are activated by the Delta (or Delta-like) and Jagged/Serrate families of membrane-bound ligands. They are transported to the plasma membrane as cleaved, but otherwise intact polypeptides. Interaction with ligand leads to two additional proteolytic cleavages that liberate the Notch intracellular domain (NICD) from the plasma membrane. The NICD translocates to the nucleus, where it forms a complex with the DNA binding protein CSL, displacing a histone deacetylase (HDAc)-co-repressor (CoR) complex from CSL. Components of an activation complex, such as MAML1 and histone acetyltransferases (HATs), are recruited to the NICD-CSL complex, leading to the transcriptional activation of Notch target genes.

|  |  |
| --- | --- |
| Reference pathway | 100% |
